# Application of direct PCR for phylogenetic analysis of *Fusarium fujikuroi* species complex isolated from rice seeds

**DOI:** 10.3389/fpls.2022.1093688

**Published:** 2023-01-13

**Authors:** Hosung Jeon, Jung-Eun Kim, Jung-Wook Yang, Hokyoung Son, Kyunghun Min

**Affiliations:** ^1^ Department of Agricultural Biotechnology, Seoul National University, Seoul, Republic of Korea; ^2^ Research Institute of Agriculture and Life Sciences, Seoul National University, Seoul, Republic of Korea; ^3^ Crop Cultivation and Environment Research Division, National Institute of Crop Science, Rural Development Administration, Suwon, Republic of Korea

**Keywords:** direct PCR, *Fusarium fujikuroi*, rice, fungal pathogen, ITS, fumonisin

## Abstract

Plant pathogenic fungi cause severe yield losses and mycotoxin contamination in crops. The precise and rapid detection of fungal pathogens is essential for effective disease management. Sequencing universal DNA barcodes has become the standard method for the diagnosis of fungal diseases, as well as for identification and phylogenetic analysis. A major bottleneck in obtaining DNA sequence data from many samples was the laborious and time-consuming process of sample preparation for genomic DNA. Here, we describe a direct PCR approach that bypasses the DNA extraction steps to streamline the molecular identification of fungal species. Using a direct PCR approach, we successfully sequenced the nuclear ribosomal internal transcribed spacer (ITS) region for the representatives of major fungal lineages. To demonstrate the usefulness of this approach, we performed a phylogenetic analysis of the *Fusarium fujikuroi* species complex, which causes bakanae (“foolish seedling”) disease of rice and mycotoxin contamination. A total of 28 candidate strains were isolated from rice seeds in the Republic of Korea, and the identity of the isolates was determined using the DNA sequence of both ITS and translation elongation factor 1-α regions. In addition, 17 *F. fujikuroi* isolates were examined for fumonisin (FB) production in rice medium using an enzyme-linked immunosorbent assay. Phylogenetic and toxigenic analyses showed that the *F. fujikuroi* strains could be distinguished into two groups: FB producers (B14-type) and non-producers (B20-type). These results will accelerate the molecular identification of fungal pathogens and facilitate the effective management of fungal diseases.

## Introduction

Pathogenic fungi pose serious threats to human health and food security. Today, the global mortality rate of fungal diseases exceeds that of breast cancer and is comparable to that of HIV (Brown et al., 2012). In agriculture, plant pathogenic fungi account for yield losses of approximately 20% worldwide, with a further 10% loss postharvest (Placinta et al., 1999; Edwards, 2004; Fisher et al., 2018). Therefore, there is an urgent need for rapid identification of fungal pathogens to facilitate effective disease management. Various methods have been developed to detect and identify pathogenic fungi (Hariharan and Prasannath, 2021). Generally, these molecular identification methods analyze the sequences of PCR-amplified universal DNA barcodes (Schoch et al., 2012; Qiu et al., 2020); this reaction requires purified genomic DNA as a template for target DNA amplification. The process of extracting DNA from fungal cells is often laborious and requires toxic chemicals (Leslie and Summerell, 2006). Therefore, it is necessary to develop a simple and reliable PCR-based approach to identify fungal pathogens without the need for DNA extraction.

The *Fusarium fujikuroi* species complex (FFSC), consisting of more than 50 phylogenetically distinct species, causes diverse diseases in agricultural crops worldwide (O’Donnell et al., 2015). FFSC species produce mycotoxins, such as fumonisin (FB) and gibberellic acid (Cerdá-Olmedo et al., 1994; Rheeder et al., 2002; Werle et al., 2020). In particular, FBs are detrimental to human health because they are associated with esophageal cancer (Yoshizawa et al., 1994; Alizadeh et al., 2012). Among the members of FFSC, *F. fujikuroi* is a causal agent of the bakanae (“foolish seedling”) disease of rice, and the most prominent symptom of the disease is hyper-elongation of seedlings (Hsieh et al., 1977; Ou, 1985; Leslie, 1995; Leslie and Summerell, 2006). However, not all infected seedlings show bakanae symptoms; some isolates cause stunting and early withering of infected seedlings (Jeon et al., 2013). Based on phylogenetic and toxigenic analyses, *F. fujikuroi* strains can be divided into two phylogenetically distinct subclades: FB producer and non-producer (Niehaus et al., 2017; Qiu et al., 2020).

Recently, *F. fujikuroi* has caused great damage to rice seedlings in the Republic of Korea, where rice is a staple food (Kim et al., 2012; Jeon et al., 2013; Choi et al., 2018). Immediate detection and identification of pathogens are important to control this disease. In this study, we developed a direct PCR approach that bypasses time-consuming DNA extraction steps. We demonstrated that direct PCR could amplify the nuclear ribosomal internal transcribed spacer (ITS) region in the representatives of major fungal lineages. We first applied a direct PCR technology for rapid identification of plant pathogenic fungi in the field. We applied this approach to the *F. fujikuroi* strains isolated from rice seeds in the Republic of Korea. The isolated *F. fujikuroi* strains had two phylogenetically distinct groups. To confirm whether mycotoxin production is phylogenetically distinct, we examined mycotoxin production in rice medium using an enzyme-linked immunosorbent assay (ELISA). Altogether, these results will accelerate the molecular identification of fungal pathogens and facilitate the effective management of fungal diseases.

## Materials and methods

### Fungal isolation

The FFSC isolates used in this study were obtained from rice seeds provided by the National Institute of Crop Science (Wanju, Republic of Korea). A total of 46 rice cultivars were examined, and 35 seeds from each cultivar were randomly chosen. All seeds were surface-sterilized with 1% sodium hypochlorite for 3 min and rinsed in sterile distilled water for 3 min; the water on the surface was removed using an autoclaved paper towel. Surface-sterilized seeds were placed on potato dextrose agar (PDA) containing 0.1% lactic acid to prevent bacterial contamination (Alakomi et al., 2000). PDA plates were incubated at 25°C for five days. To isolate *F. fujikuroi* strains, 293 candidate strains were initially selected based on colony morphology. We selected the final 28 strains based on morphological characteristics, such as conidial shapes. All strains grown on PDA were stored in 20% glycerol solution at -80°C.

The representative fungal strains used in this study were provided by the Rural Development Administration (Jeonju, Republic of Korea), Korean Collection for Type Cultures (Jeongeup, Republic of Korea), Fungal Plant Pathology Lab. (Seoul National University, Seoul, Republic of Korea), Chung-Ang University (Seoul, Republic of Korea), Soonchunhyang University (Asan, Republic of Korea), and Chonnam National University (Gwangju, Republic of Korea). Detailed information about all strains used in this study is listed in **Table S1**.

### Direct PCR amplification and sequencing

The oligonucleotide primer pairs ITS4 (5′-TCCTCCGCTTATTGATATGC-3′)/ITS5 (5′- GGAAGTAAAAGTCGTAACAAGG-3′) and EF1T (5′-ATGGGTAAGGAGGACAAGAC-3′)/EF2T (5′-GGAAGTACCAGTGATCATGTT-3′) were used for the amplification of the ITS and *TEF-1α* regions, respectively (O'Donnell et al., 1998). All oligonucleotide primer sets were synthesized at an oligonucleotide synthesis facility (Bioneer, Daejeon, Republic of Korea). Amplification reactions were performed in a total volume of 20 μl AccuPower PCR Premix kit (Bioneer), containing 19 μl distilled water and 0.5 μl of each primer (20 μM). A small amount of mycelium grown on PDA was added to the reaction mixture using sterilized pipette tips. We conducted additional steps to facilitate the amplification of the target regions. The reaction mixture was heat shocked for 3 min at 95°C and cooled to 25°C. The PCR tubes were vortexed vigorously for 1 min to break down the cells. The thermal cycle was as follows: an initial 1-min denaturation at 95°C, followed by 40 cycles of 30-sec denaturation at 95°C, 1-min annealing at 53°C and 56°C for ITS and *TEF-1α*, respectively, 1 min at 72°C, followed by a final extension of 3 min at 72°C. The amplified products (5 μl) and 100 bp Plus DNA Ladder (Bioneer) were loaded on a 1.5% (w/v) agarose gel with Tris-acetate-EDTA (TAE) buffer. The products were purified using the α+ SolutionTM GEL/PCR Purification Kit (Alphagen, Changzhi, Taiwan) and submitted to the Bioneer sequencing service. The primers used for sequencing were the same as those used for amplification. DNA sequences were used to conduct a BLAST search against the NCBI GenBank database for species identification. Conventional PCR was performed on strains for which direct PCR did not work. For conventional PCR, fungal genomic DNA was extracted using the CTAB method (Leslie and Summerell, 2006). We added 0.5 μl of the genomic DNA to the total volume of 20 μl AccuPower PCR Premix (Bioneer). The thermal cycle for conventional PCR was the same as that for direct PCR; however, there was no additional heat shock step. The purified PCR products were submitted to the Bioneer sequencing service.

To determine their pathotypes, the *F. fujikuroi* isolates were subjected to PCR using the primer pairs B14J06375F2/B14J05375R2 and B20J12141F2/B20J12141R2 for B14- and B20-type, respectively (Niehaus et al., 2017). The *F. fujikuroi* B14 strain, but not the B20 strain, reportedly produces FBs; hence, we used B14 and B20 strains as controls (Niehaus et al., 2017). We used direct PCR protocols, as described above, with a modification of the annealing temperature to 60°C. The amplified products were loaded onto 1.5% (w/v) agarose gel.

### Toxigenic analysis

To induce mycotoxins, such as FBs, from *F. fujikuroi*, we cultured 17 *F. fujikuroi* isolates on a rice medium. In brief, 1.5 g of rice and 1 ml of distilled water were autoclaved in 8 ml vials. The rice medium was inoculated with the PDA agar blocks on which the isolates were grown. The vials were incubated for three weeks at 25°C in the dark. To extract mycotoxins, the 1.5 g of rice cultures were freeze-dried overnight and ground into a powder. Mycotoxins were extracted with 3 ml of 85% methanol with vigorous shaking for 5 min. The samples were centrifuged at 470 ×g at 4°C for 5 min, and 500 μl of the supernatant was transferred into a new tube. The extract was filtered through a Millex syringe filter (hydrophilic PTFE membrane, 0.45 μm pore size), and filtrates were stored at 4°C until analysis. The level of FBs was measured using a commercial ELISA kit (AgraQuant Fumonisin Kit; Romer Labs, Austria), according to the manufacturer’s instructions. The sample was diluted 100,000 times in distilled water to ensure that the FB concentration was within the detection range. The toxigenic analysis was repeated three times.

### Data analysis

The *TEF-1α* sequence of FFSC was used for the phylogenetic analysis of FFSC. Additional sequences of FFSC strains (MN696157, MN696156, MN695931, and MN386748.1) were obtained from GenBank (Qiu et al., 2020). The *TEF-1α* sequence of *F. oxysporum* strain (KX253985.1) was also obtained from GenBank. The DNA sequences were first trimmed using SeqMan software (DNASTAR Inc., Madison, USA) and then aligned and used to construct phylogenetic trees using MEGA11 software. We used the neighbor-joining (NJ) method for constructing phylogenetic trees and evaluated the topology of the trees by the bootstrap method based on 1000 replications. Analyses of mycotoxin production using ELISA kits were performed using a Synergy HTX microplate reader (BioTek, Winooski, USA) to measure absorbance. The absorbance value was analyzed to calculate the FB concentration in the rice medium using Microsoft Excel (Microsoft, Redmond, USA) and GraphPad Prism 8 (GraphPad Software, San Diego, USA), following the manufacturer’s instructions. Scientific illustrations were created using BioRender (Biorender.com), and other figures were generated using Microsoft PowerPoint (Microsoft).

## Results

### Fungal identification without genomic DNA extraction

**Figure 1:**
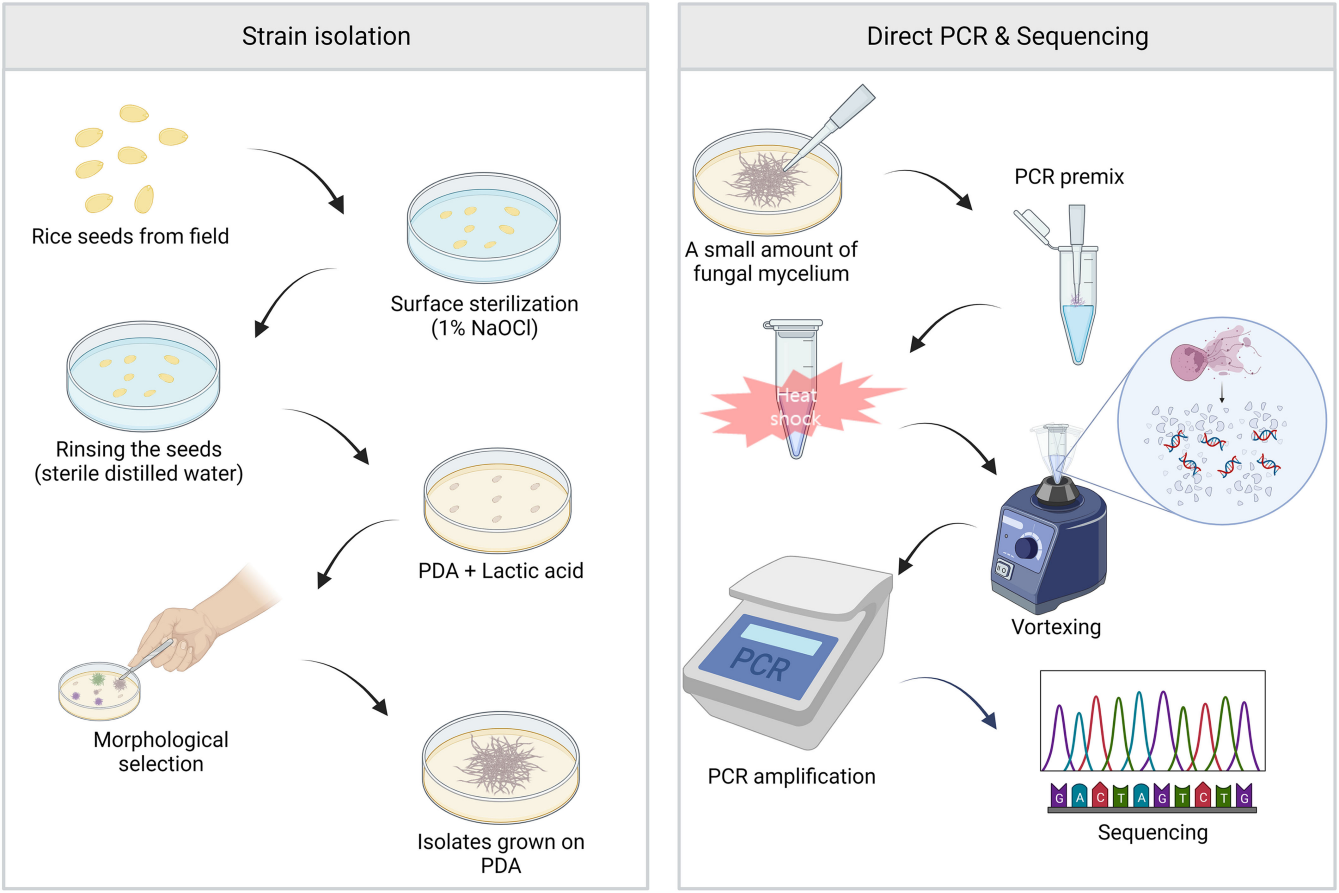
The main workflow of this study. Left panel, isolation of fungal strains. The surface of rice seeds collected from the field was sterilized with 1% of sodium hypochlorite for 3 min, followed by rinsing with sterile distilled water for 3 min. The seeds were placed on potato dextrose agar (PDA) containing lactic acid, and the plate was incubated at 25°C for five days. The candidate strains of FFSC were isolated based on their morphological characters. Right panel, direct PCR and sequencing. A small amount of fungal mycelium was added to the PCR premixture using pipette tips. To amplify target regions, modified PCR processes involving additional heat shock and vortexing steps prior to the thermocycling were carried out, and the amplified products were sequenced.

**Figure 2:**
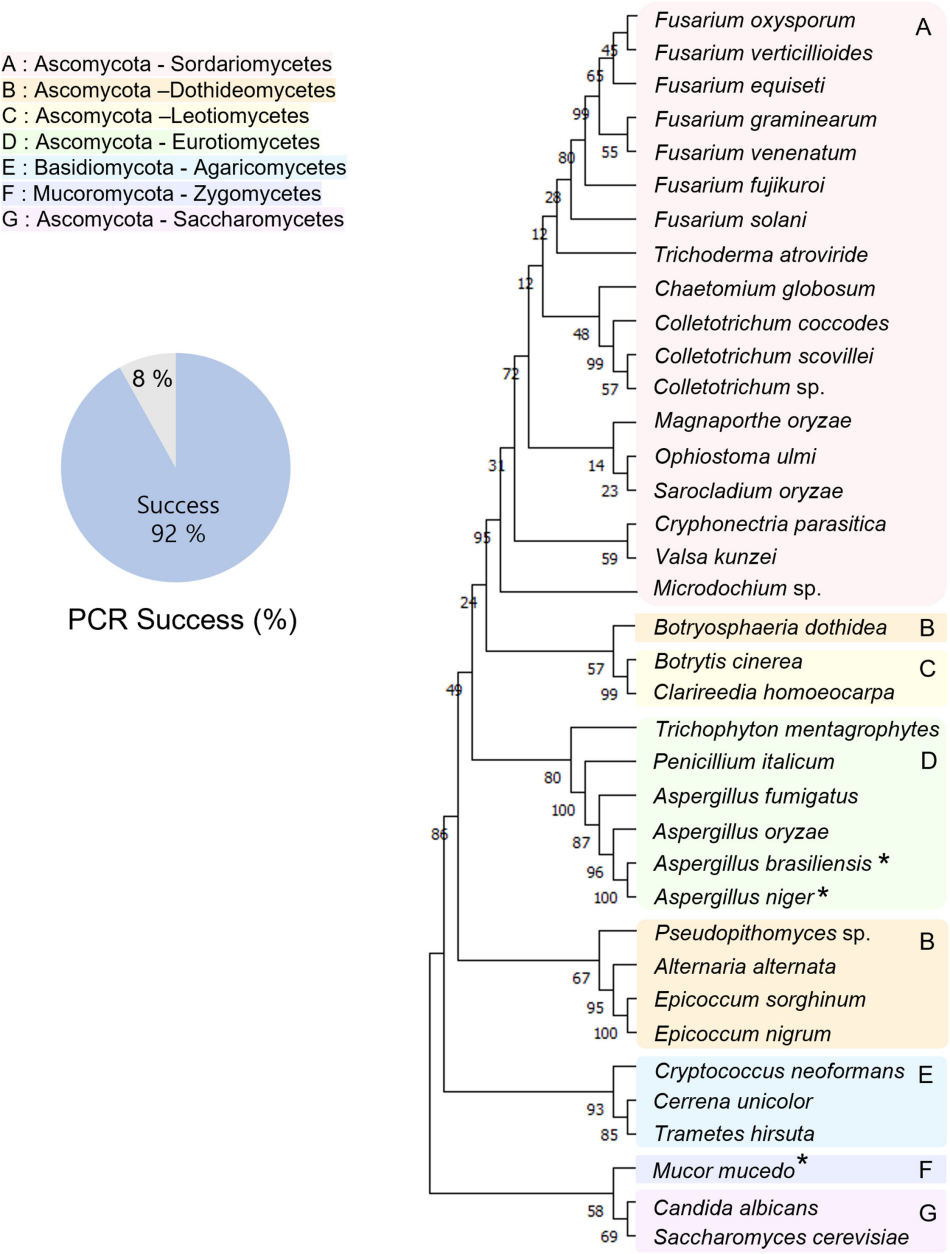
Phylogenetic tree of representatives of major fungal lineages. The tree is inferred from ITS sequences obtained by the direct PCR approach. The asterisks indicate that the sequences of these strains were obtained by the conventional PCR method involving genomic DNA extraction steps. The representatives of the major fungal lineages are clustered by the classes. A pie chart indicating the PCR success rate shows that the direct PCR approach is reliable and applicable. **A**: Sordariomycetes, **B**: Dothideomycetes, **C**: Leotiomycetes, **D**: Eurotiomycetes, **E**: Agaricomycetes, **F**: Zygomycetes, **G**: Saccharomycetes. Species of the same color indicate they are in the same class.

Rapid detection and identification of fungal pathogens are important. Thus, we used a direct PCR approach for the detection and identification of fungal species. The representatives of major fungal lineages were used to demonstrate the application of PCR to various fungal species. The experimental scheme is shown in **Figure 1**. Using the direct PCR approach, we amplified the ITS region of representatives of major fungal lineages (**Figure 2**). Among the 37 fungal strains, the ITS region of 34 fungal strains (92%) was successfully amplified using direct PCR. However, direct PCR could not amplify the ITS region of three fungal strains: *Aspergillus brasiliensis*, *Aspergillus niger*, and *Mucor mucedo*. Genomic DNA was extracted, and the ITS region was amplified as previously described (Qiu et al., 2020). The ITS sequences of all the fungal strains analyzed matched the species identification (**Table S1**). The ITS sequences of the 37 strains were used to construct a phylogenetic tree (**Figure 2**).

### Phylogenetic analysis of *F. fujikuroi* species complex

**Figure 3:**
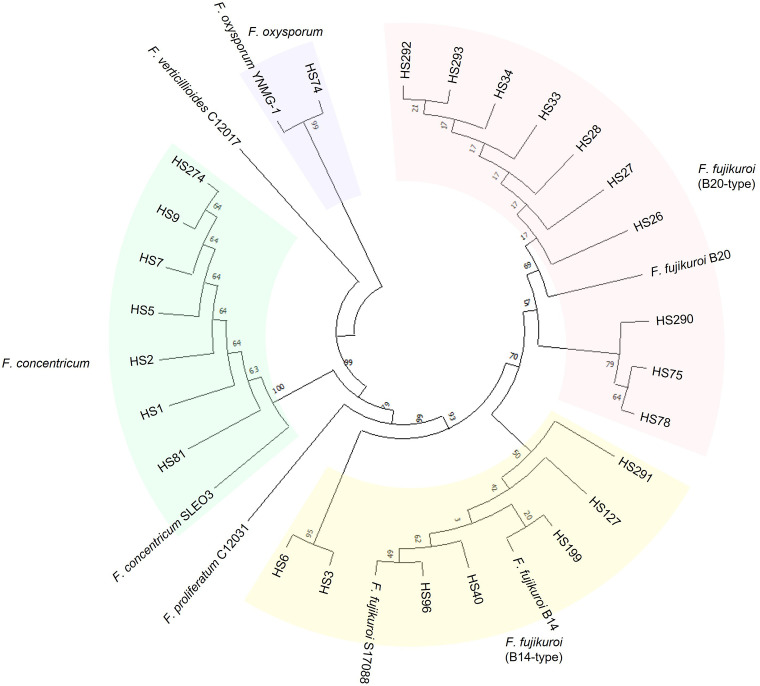
A phylogenetic tree of the FFSC strains inferred from the *TEF-1α* gene sequence. In the phylogenetic tree, *F. concentricum*, *F. verticillioides*, *F. proliferatum*, and two types of *F. fujikuroi* strains belong to FFSC. The *F. fujikuroi* strains are distinguished into two groups: B14-type and B20-type. Two strains of *F. fujikuroi*, B14 and B20, known as FB producer and non-producer, respectively, were used as references. *TEF-1α* gene sequences of four FFSC strains (C12017, C12031, S17088, and SLEO3) and the *F. oxysporum* strain (YNMG-1) were obtained from GenBank. Strains of the same color indicate they are in the same phylogenetic group.

**Figure 4:**
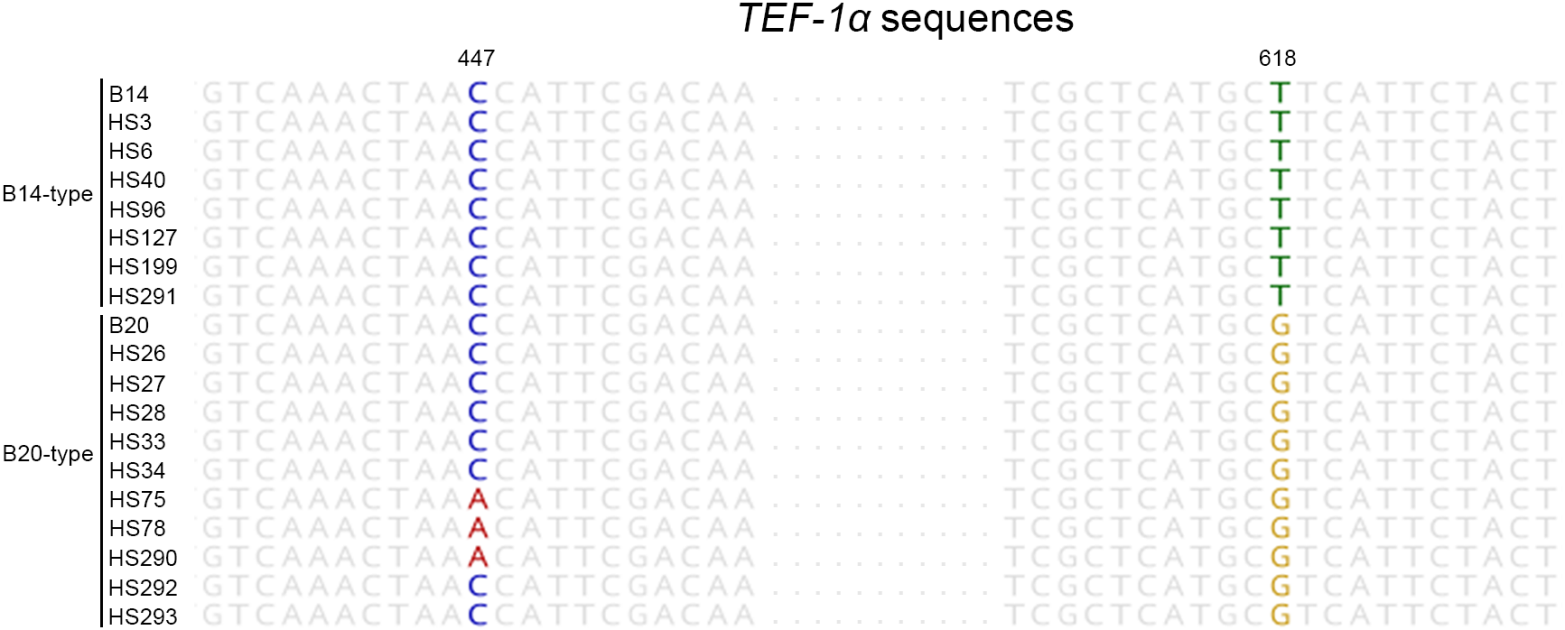
The nucleotide differences among aligned *TEF-1α* gene sequences of *F. fujikuroi* strains. The aligned sequences show that the B14- and B20-type of *F. fujikuroi* have thymine and guanine at nucleotide position 618, respectively. Also, B14-type strains have cytosine at nucleotide position 447, however, the B20-type of *F. fujikuroi* has either adenine or cytosine at this position.

We demonstrated the usefulness of the direct PCR approach by applying it to the identification of field isolates. FFSC, an important component of the genus *Fusarium*, consists of distinct species, such as *F. fujikuroi*, *F. proliferatum*, and *F. verticillioides* (Qiu et al., 2020). These strains can cause devastating cereal diseases. Therefore, we isolated FFSC strains from the seeds of 46 rice cultivars harvested in Jeonju and Miryang in the Republic of Korea (**Figure 1**). The 28 FFSC candidate strains formed white mycelia that turned violet or orange with age on PDA. This pigmentation was the same as that in previous reports (Leslie and Summerell, 2006). For molecular identification, we used both the ITS and *TEF-1α* regions because the FFSC strains were indistinguishable in their ITS sequences. The target regions were amplified using a direct PCR approach, and the amplified products were directly sequenced (**Supplementary Figure S1**). Based on sequence similarity, 28 candidate strains were identified as *F. fujikuroi* (17 strains), *F. concentricum* (7 strains), *F. incarnatum* (1 strain), *F. oxysporum* (1 strain), *F. commune* (1 strain), and *Colletotrichum* sp. (1 strain). Interestingly, 7 out of 17 *F. fujikuroi* strains were isolated from a rice cultivar, Chamdongjin (**Supplementary Figure S2**). The other 10 strains were isolated from a variety of rice cultivars: Hyunpoom, Haepoom, Boramchal, Shindongjin, Mokyang, Alchanmi, Saebonghwang, and Mipoom. A phylogenetic tree was constructed based on the sequences of the *TEF-1α* gene (**Figure 3**). In the phylogenetic tree, *F. fujikuroi* strains were divided into two groups, B14 and B20. The B14-type group includes the B14 strain, known to produce FB (Niehaus et al., 2017). In contrast, the B20 strain in the B20-type group did not produce FB. In *F. fujikuroi*, multiple alignments of the *TEF-1α* sequences revealed that B14-type *F. fujikuroi* strains have thymine at nucleotide position 618 of the *TEF-1α* gene sequences. In contrast, B20-type *F. fujikuroi* strains have guanine at this site (**Figure 4**).

### Analysis of mycotoxin production

Seventeen *F. fujikuroi* strains were analyzed to examine their FB-producing ability. The strains were grown in a rice medium to induce FB production. Using ELISA, FBs were detected in the seven *F. fujikuroi* strains that clustered with the B14 strain (**Table 1**). The FB concentration was very high, ranging from 46 to 205 mg/g, in the rice medium. However, FBs were not detected in the 10 strains that clustered with the B20-type group. This indicates that FB production correlates with phylogenetic group.

**Table 1:** Levels of fumonisin produced by *F. fujikuroi* isolates measured using ELISA.

Species	Isolate	FB (mg/g)	Host
*F. fujikuroi*	HS3	46 ± 10	Rice
	HS6	53 ± 13	Rice
	HS26	ND	Rice
	HS27	ND	Rice
	HS28	ND	Rice
	HS33	ND	Rice
	HS34	ND	Rice
	HS40	72 ± 20	Rice
	HS75	ND	Rice
	HS78	ND	Rice
	HS96	117 ± 25	Rice
	HS127	122 ± 33	Rice
	HS199	85 ± 30	Rice
	HS290	ND	Rice
	HS291	205 ± 99	Rice
	HS292	ND	Rice
	HS293	ND	Rice

ND, not detected; FB, fumonisin.

A previous study reported a relationship between mycotoxin production in *F. fujikuroi* isolates and the secondary metabolite genes *PKS51* and *NRPS31* (Niehaus et al., 2017). Therefore, we examined 17 *F. fujikuroi* strains using pathotype-specific primer pairs amplifying *PKS51* and *NRPS31* fragments (**Supplementary Figure S3**). The PCR genotyping showed that the *F. fujikuroi* strains were divided into two groups: B14-type and B20-type. *PKS51* primer pair could successfully amplify the gene in *F. fujikuroi* strains producing FB, while the PCR product of *NRPS31* was obtained from FB-non-producing *F. fujikuroi* strains. However, two strains, HS96 and HS291, had no PCR amplification although we used both direct PCR and genomic DNA extraction. They produced FB in rice medium and were grouped with the B14-type strain in the phylogenetic analysis (**Table 1** and **Figure 3**).

## Discussion

Sequencing the DNA barcode has been widely used to identify fungal pathogens (Alshahni et al., 2009; Schoch et al., 2012; Qiu et al., 2020). The differences in the methods can significantly affect the efficiency of identification. In this study, we describe an optimized approach for the identification of fungal species. We compared our approach to previous methods for fungal identification (**Table 2**). The methods used by Schoch et al. and Qiu et al. required sample preparation for genomic DNA (Schoch et al., 2012; Qiu et al., 2020). To prepare DNA samples, the fungal strains were cultured for a few days and harvested before genomic DNA extraction. In addition, the DNA samples extracted by Schoch et al. required an additional purification step before the PCR amplification. The method used by Alshahni et al. bypassed the requirement for genomic DNA extraction. Instead, this method involved steps that lyse fungal cell walls using a lysis buffer. Walch et al. added bovine serum albumin (BSA) to PCR mixture for a direct fungal colony PCR (Walch et al., 2016). However, our direct PCR method bypasses the need for genomic DNA extraction and additional reagents such as proteinase K or BSA. The amplified products were sequenced directly without molecular cloning. Using direct PCR, we amplified the ITS region of representatives of major fungal lineages, and the amplified products were sequenced directly (**Figure 2**). In addition, we isolated FFSC strains in the field and amplified the ITS and *TEF-1α* regions using direct PCR (**Supplementary Figure S1**). The high PCR success rate and sequence quality demonstrated the reliability and applicability of this method. We believe that direct PCR streamlines the process of molecular identification of fungal pathogens.

**Table 2:** Comparison of PCR approaches for fungal identification.

Attribute	Schoch et al.	Qiu et al.	AlShahni et al.	Jeon et al.
DNA extraction free	−	−	+	+
No need for cell lysis	+/−	−	−	+
Direct sequencing of PCR products	+	+	−	+
Efficient and rapid species identification	−	−	+	+

In the previous report, Lee infected rice seedlings with *F. fujikuroi* and measured resistance to the disease in the lab (Lee, 2022). The author divided 66 rice cultivars into 3 groups based on resistance to bakanae disease: resistant, intermediate, and susceptible. In this study, we isolated *F. fujikuroi* strains from rice seeds harvested from rice paddy fields. Interestingly, most of the *F. fujikuroi* strains were isolated from the susceptible group, and no strain from intermediate and resistant groups. For example, 7 out of 17 *F. fujikuroi* strains were isolated from Chamdongjin, which are reportedly susceptible to bakanae disease (**Supplementary Figure S2**). The remaining 10 strains were isolated from other cultivars that were mostly susceptible to *F. fujikuroi*. Our study is significant because we confirmed the laboratory result in a real agricultural setting.

In this study, the *F. fujikuroi* isolates were distinguished into two groups (B14-type and B20-type) based on *TEF-1α* gene sequences (**Figure 3**). Thus, we tested the relationship between the phylogenetic group and FB production in *F. fujikuroi*. Using ELISA kits, FBs were detected in all B14-type strains but not in any of the B20-type strains (**Table 1**). The previous study found nucleotide differences in the *TEF-1α* gene sequences between B14-type and B20-type *F. fujikuroi* strains (Qiu et al., 2020). In the *TEF-1α* gene sequences, all B14-type strains had cytosine and thymine at nucleotide positions 447 and 618, respectively. In contrast, all B20-type strains contained adenine and guanine at these positions. In our study, B14- and B20-type strains had thymine and guanine at nucleotide position 618, respectively (**Figure 4**). However, we found that nucleotide position 447 of *TEF-1α* gene did not match phylogenetic group and FB production of the *Fusarium fujikuroi* strains. Eight B20-type strains (HS26, HS27, HS28, HS33, HS34, HS292, HS293, and B20) had cytosine at nucleotide position 447. This result is different from the previous report that FB non-producers (B20-type) had adenine at nucleotide position 447 (Qiu et al., 2020). This indicates that the *F. fujikuroi* populations have higher genetic variation than previously reported.

In comparative genome analysis, *PKS51* gene cluster was present in FB-producing *F. fujikuroi* stains but absent in FB non-producers (Niehaus et al., 2017). In contrast, *NRPS31* gene cluster was absent in the FB producers but present in the FB non-producers. Although the *PKS51* and *NRPS31* genes are not in the FB biosynthetic pathway, researchers have used their genotypes to predict FB production. (Niehaus et al., 2017; Choi et al., 2018; Qiu et al., 2020). Based on the previous studies, we performed direct PCR genotyping to predict FB production (**Supplementary Figure S3**). However, in this study, PCR amplification of *PKS51* was not successful in some FB-producing strains. This inconsistency has been previously reported (Choi et al., 2018). These results indicate high genetic variation of *PKS51* in *F. fujikuroi* populations. In addition, the product and function of Pks51 are unknown, and it is not clear how it relates to FB production. Nrps31 synthesizes apicidin F, but apicidin F is not related to FB biosynthesis. Taken together, these results raise the question whether genotypes of *PKS51* and *NRPS31* can accurately predict FB production. We suggest that phylogenetic analysis of the *TEF-1α* sequence is a better way to predict FB production.

In summary, we demonstrated that direct PCR is an effective way to identify fungal species. Direct PCR was performed for major fungal species and field isolates, demonstrating the usefulness of this approach. The fact that DNA amplification can occur directly from fungal mycelia has several implications in mycology. Therefore, we suggest that direct PCR may have additional applications. For example, in the field, it could be possible to isolate fungal mycelia or spores from infected plants and perform direct PCR without culturing the fungus. This method will be useful for the identification of fungal species that are difficult to culture. In addition, one could perform PCR genotyping to screen for the right mutants without genomic DNA extraction. This can accelerate future functional analyses of genes in fungi. We believe that this direct PCR system will contribute to advancements in molecular studies of filamentous fungi.

## Data availability statement

The datasets presented in this study can be found in online repositories. The names of the repository/repositories and accession number(s) can be found in the article/**Supplementary Material**.

## Author contributions

HJ, J-EK, J-WY, HS, and KM conceived and designed the experiments. HJ and KM conducted the experiments and analyzed the data. HJ, J-EK, HS, and KM wrote and edit the manuscript. All authors contributed to the article and approved the submitted version.

## Supplementary material

Supplementary Figure 1 Gel electrophoresis images of amplified products of FFSC strains using the direct PCR approach. To amplify target regions (ITS and *TEF-1α*), we used primer pairs ITS4/ITS5 and EF1T/EF2T for direct PCR, and the amplified products were loaded on a 1.5% (w/v) agarose gel. The target regions and size of amplicons are shown on the left.

Supplementary Figure 2 Incidence of the *F. fujikuroi* strains from Korean rice cultivars. Seventeen *F. fujikuroi* strains were recovered from rice seeds. Among the 17 strains, 7 were isolated from Chamdongjin. The other strains were isolated from other cultivars (Haepoom, Hyunpoom, Boramchal, Shindongjin, Saebonghwang, Mokyang, Mipoom, and Alchanmi).

Supplementary Figure 3 Direct PCR amplification using primer pairs B14J06375F2/B14J05375R2 and B20J12141F2/B20J12141R2 for B14-type and B20-type, respectively. The primer pairs for B14-type and B20-type were derived from *PKS51* and *NRPS31*, respectively.
