# Supplementary material for: Application of direct PCR for phylogenetic analysis of Fusarium fujikuroi species complex isolated from rice seeds
Source: Front Plant Sci. 2023 Jan 13;13:1093688. doi: 10.3389/fpls.2022.1093688 (PMC9880262; doi:10.3389/fpls.2022.1093688)
Supplement: Supplementary file 1 [file Table_1.docx]

Supplementary Material

# Supplementary table

**Table S1.** Lists of all strains used in this study.

| Species | Strain number | Origin |
| --- | --- | --- |
| *Alternaria alternata* | KCTC6005 | Korean Collection for Type Cultures (Jeongeup, Republic of Korea) |
| *Aspergillus brasiliensis* | ATCC16404 | Korean Collection for Type Cultures (Jeongeup, Republic of Korea) |
| *Aspergillus fumigatus* | KACC41016 | Rural Development Administration (Jeonju, Republic of Korea) |
| *Aspergillus niger* | KCTC6317 | Korean Collection for Type Cultures (Jeongeup, Republic of Korea) |
| *Aspergillus oryzae* | KACC44823 | Rural Development Administration (Jeonju, Republic of Korea) |
| *Botryosphaeria dothidea* | P1-12-5-2-51 | Chonnam National University (Gwangju, Republic of Korea) |
| *Botrytis cinerea* | P1-12-5-2-54 | Chonnam National University (Gwangju, Republic of Korea) |
| *Candida albicans* | KCTC7965 | Korean Collection for Type Cultures (Jeongeup, Republic of Korea) |
| *Cerrena unicolor* | P1-16-2-1-51 | - |
| *Chaetomium globosum* | KCTC6059 | Korean Collection for Type Cultures (Jeongeup, Republic of Korea) |
| *Clarireedia homoeocarpa* | P1-12-5-4-10 | Chonnam National University (Gwangju, Republic of Korea) |
| *Colletotrichum coccodes* | P1-12-5-2-57 | Chonnam National University (Gwangju, Republic of Korea) |
| *Colletotrichum scovillei* | P1-17-2-4-2 | Spicy pepper |
| *Colletotrichum* sp. | HS10 | Rice (Jeonju, Republic of Korea, Namchan, 2022) |
| *Cryphonectria parasitica* | P1-12-5-2-60 | Chonnam National University (Gwangju, Republic of Korea) |
| *Cryptococcus neoformans* | P1-17-4-3-23 | Chung Ang University (Seoul, Republic of Korea) |
| *Epicoccum nigrum* | KACC40313 | Rural Development Administration (Jeonju, Republic of Korea) |
| *Epicoccum sorghinum* | HS109 | Rice (Miryang, Republic of Korea, Samgwang, 2022) |
| *Fusarium equiseti* | HS56 | Rice (Jeonju, Republic of Korea, Shindongjin, 2022) |
| *Fusarium fujikuroi* | KACC45818 | Rural Development Administration (Jeonju, Republic of Korea) |
| *Fusarium graminearum* | Z-3639 | Bowden and Leslie, 1999 |
| *Fusarium oxysporum* | P1-12-5-2-63 | Chonnam National University (Gwangju, Republic of Korea) |
| *Fusarium solani* | P1-13-2-3-13 | - |
| *Fusarium venenatum* | ATCC20334 | American Type Culture Collection (Virginia, USA) |
| *Fusarium verticillioides* | P1-12-5-2-69 | Chonnam National University (Gwangju, Republic of Korea) |
| *Magnaporthe oryzae* | P1-18-1-4-32 | Fungal Plant Pathology Lab. (Seoul National University, Seoul, Republic of Korea) |
| *Microdochium* sp. | HS29 | Rice (Jeonju, Republic of Korea, Chamdongjin, 2022) |
| *Mucor mucedo* | KACC46082 | Rural Development Administration (Jeonju, Republic of Korea) |
| *Ophiostoma ulmi* | P1-12-5-2-72 | Chonnam National University (Gwangju, Republic of Korea) |
| *Penicillium italicum var italicum* | KCTC6437 | Korean Collection for Type Cultures (Jeongeup, Republic of Korea) |
| *Pseudopithomyces* sp. | HS156 | Rice (Jeonju, Republic of Korea, Chuchung, 2022) |
| *Saccharomyces cerevisiae* | KCTC 7913 | Korean Collection for Type Cultures (Jeongeup, Republic of Korea) |
| *Sarocladium oryzae* | P1-18-1-4-33 | Fungal Plant Pathology Lab. (Seoul National University, Seoul, Republic of Korea) |
| *Trametes hirsuta* | P1-16-2-1-50 | - |
| *Trichoderma atroviride* | KACC40774 | Rural Development Administration (Jeonju, Republic of Korea) |
| *Trichophyton mentagrophytes* | P1-12-5-4-28 | - |
| *Valsa kunzei* | P1-12-5-4-13 | Chonnam National University (Gwangju, Republic of Korea) |
| *Fusarium concentricum* | HS1 | Rice (Jeonju, Republic of Korea, Hyunpoom, 2022) |
| *Fusarium concentricum* | HS2 | Rice (Jeonju, Republic of Korea, Hyunpoom, 2022) |
| *Fusarium fujikuroi* | HS3 | Rice (Jeonju, Republic of Korea, Hyunpoom, 2022) |
| *Fusarium concentricum* | HS5 | Rice (Jeonju, Republic of Korea, Hyunpoom, 2022) |
| *Fusarium fujikuroi* | HS6 | Rice (Jeonju, Republic of Korea, Hyunpoom, 2022) |
| *Fusarium concentricum* | HS7 | Rice (Jeonju, Republic of Korea, Hyunpoom, 2022) |
| *Fusarium concentricum* | HS9 | Rice (Jeonju, Republic of Korea, Hyunpoom, 2022) |
| *Fusarium fujikuroi* | HS26 | Rice (Jeonju, Republic of Korea, Chamdongjin, 2022) |
| *Fusarium fujikuroi* | HS27 | Rice (Jeonju, Republic of Korea, Chamdongjin, 2022) |
| *Fusarium fujikuroi* | HS28 | Rice (Jeonju, Republic of Korea, Chamdongjin, 2022) |
| *Fusarium fujikuroi* | HS33 | Rice (Jeonju, Republic of Korea, Chamdongjin, 2022) |
| *Fusarium fujikuroi* | HS34 | Rice (Jeonju, Republic of Korea, Chamdongjin, 2022) |
| *Fusarium fujikuroi* | HS40 | Rice (Jeonju, Republic of Korea, Boramchal, 2022) |
| *Fusarium commune* | HS57 | Rice (Jeonju, Republic of Korea, Shindongjin, 2022) |
| *Fusarium oxysporum* | HS74 | Rice (Jeonju, Republic of Korea, Haepoom, 2022) |
| *Fusarium fujikuroi* | HS75 | Rice (Jeonju, Republic of Korea, Haepoom, 2022) |
| *Fusarium fujikuroi* | HS78 | Rice (Jeonju, Republic of Korea, Haepoom, 2022) |
| *Fusarium concentricum* | HS81 | Rice (Jeonju, Republic of Korea, Saenuri, 2022) |
| *Fusarium fujikuroi* | HS96 | Rice (Jeonju, Republic of Korea, Mipoom, 2022) |
| *Fusarium fujikuroi* | HS127 | Rice (Miryang, Republic of Korea, Alchanmi, 2022) |
| *Fusarium fujikuroi* | HS199 | Rice (Miryang, Republic of Korea, Mokyang, 2022) |
| *Fusarium incarnatum* | HS238 | Rice (Miryang, Republic of Korea, Haedeul, 2022) |
| *Fusarium concentricum* | HS274 | Rice (Miryang, Republic of Korea, Ohdae, 2022) |
| *Fusarium fujikuroi* | HS290 | Rice (Jeonju, Republic of Korea, Shindongjin, 2022) |
| *Fusarium fujikuroi* | HS291 | Rice (Jeonju, Republic of Korea, Saebonghwang, 2022) |
| *Fusarium fujikuroi* | HS292 | Rice (Jeonju, Republic of Korea, Chamdongjin, 2022) |
| *Fusarium fujikuroi* | HS293 | Rice (Jeonju, Republic of Korea, Chamdongjin, 2022) |
| *Fusarium fujikuroi* B14 | P1-15-2-1-30 | Soonchunhyang University (Asan, Republic of Korea) |
| *Fusarium fujikuroi* B20 | P1-15-2-1-31 | Soonchunhyang University (Asan, Republic of Korea) |
