# Supplementary figures and images for: Application of direct PCR for phylogenetic analysis of Fusarium fujikuroi species complex isolated from rice seeds
Source: Front Plant Sci. 2023 Jan 13;13:1093688. doi: 10.3389/fpls.2022.1093688 (PMC9880262; doi:10.3389/fpls.2022.1093688)

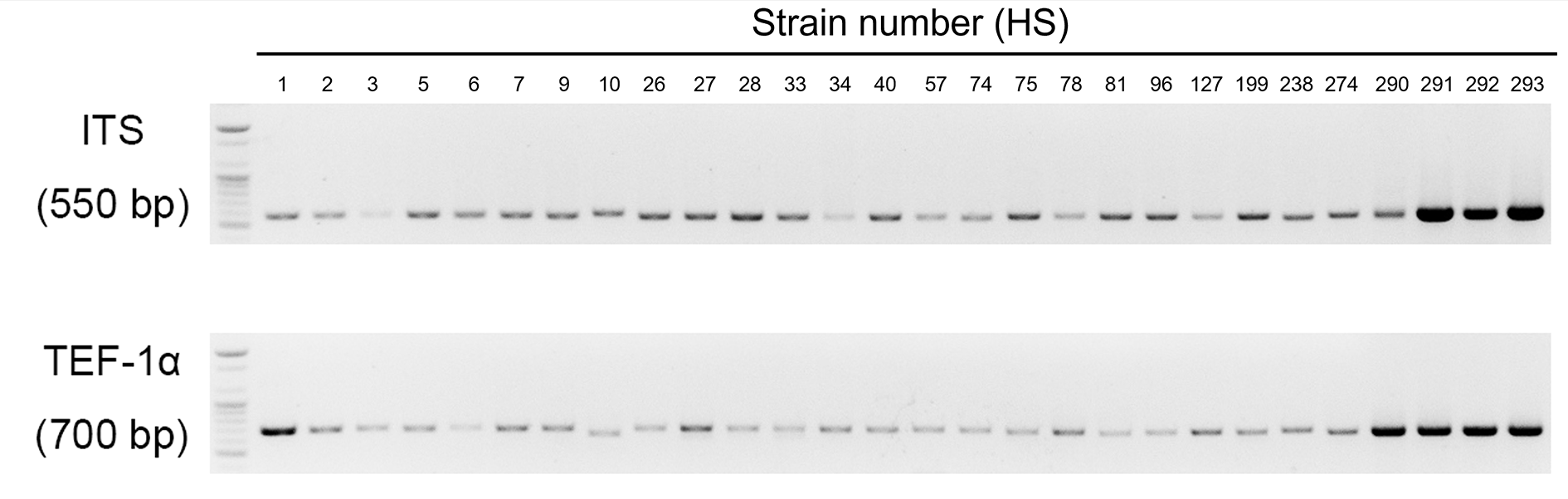

Supplement: Supplementary Figure 1 — Gel electrophoresis images of amplified products of FFSC strains using the direct PCR approach. To amplify target regions (ITS and TEF-1α), we used primer pairs ITS4/ITS5 and EF1T/EF2T for direct PCR, and the amplified products were loaded on a 1.5% (w/v) agarose gel. The target regions and size of amplicons are shown on the left. [file Image_1.tif]

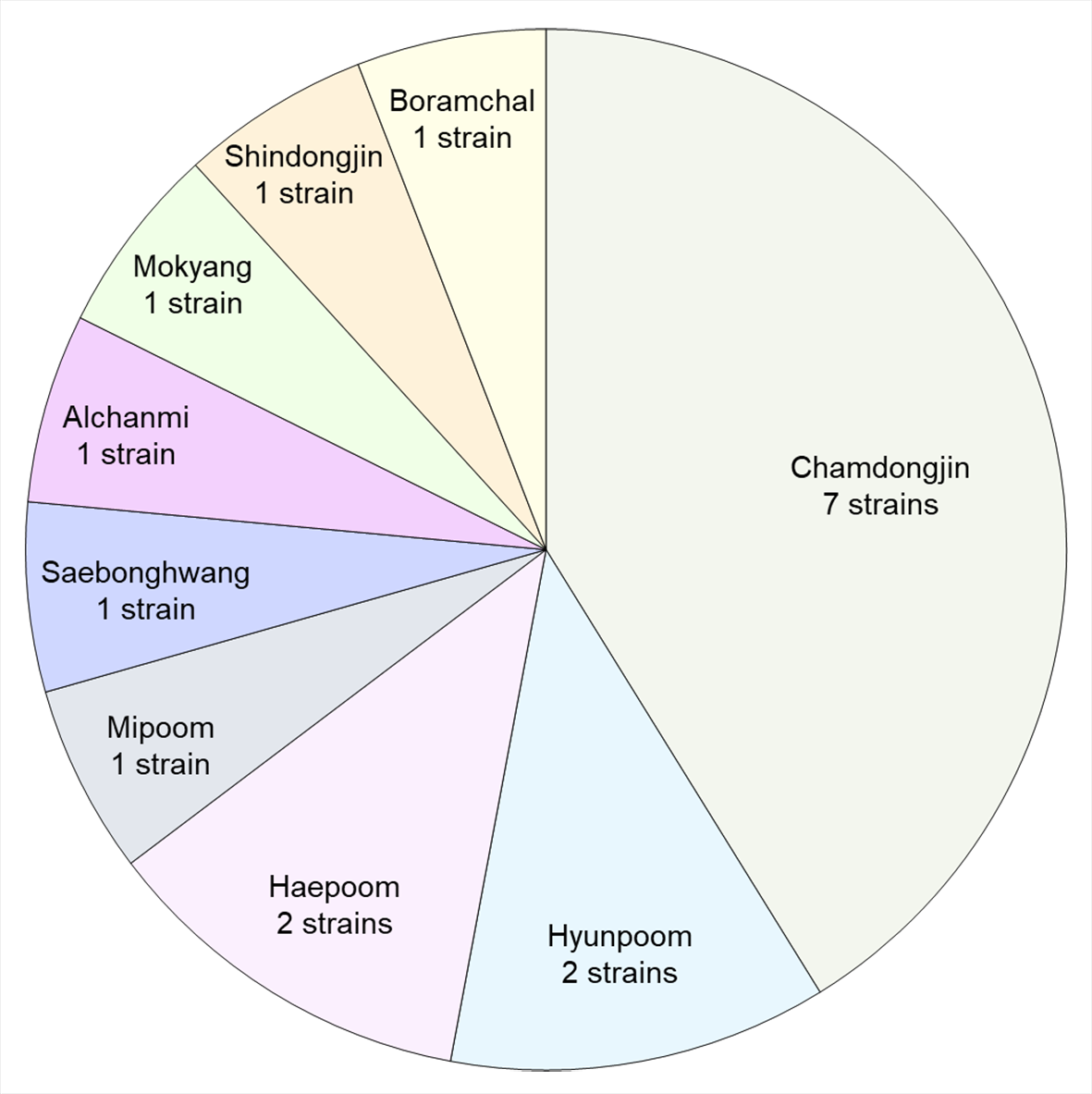

Supplement: Supplementary Figure 2 — Incidence of the F. fujikuroi strains from Korean rice cultivars. Seventeen F. fujikuroi strains were recovered from rice seeds. Among the 17 strains, 7 were isolated from Chamdongjin. The other strains were isolated from other cultivars (Haepoom, Hyunpoom, Boramchal, Shindongjin, Saebonghwang, Mokyang, Mipoom, and Alchanmi). [file Image_2.tif]

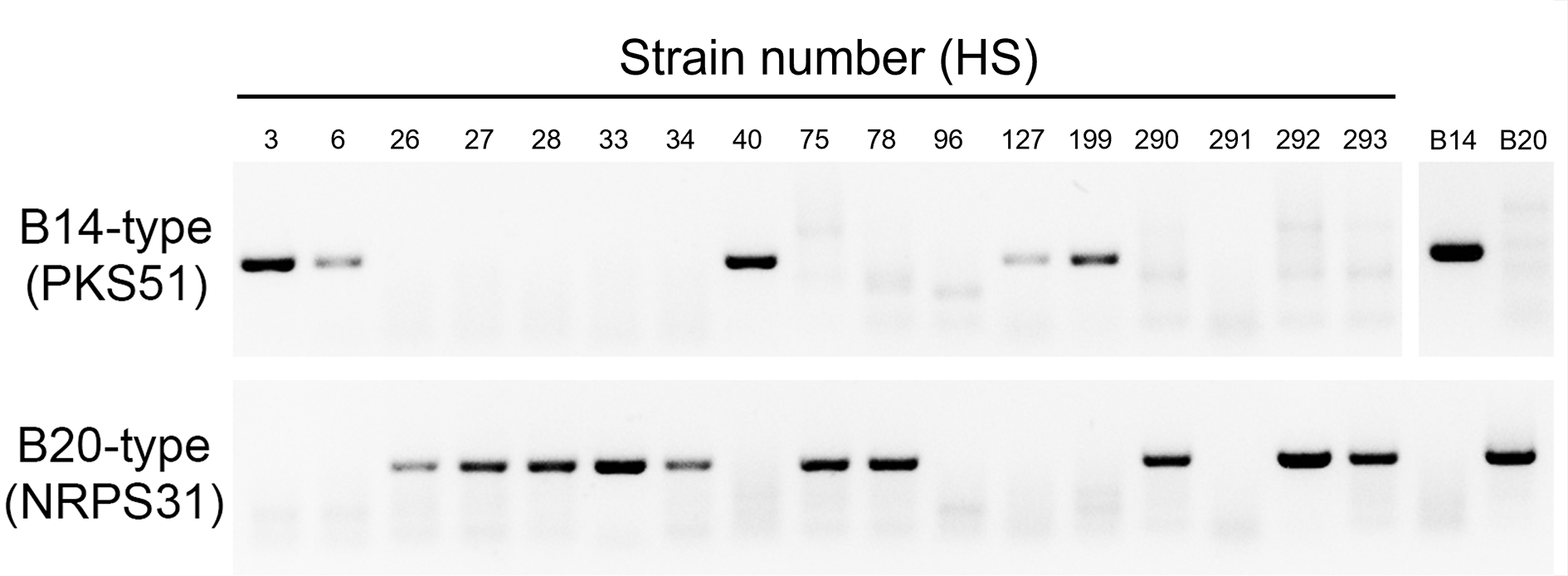

Supplement: Supplementary Figure 3 — Direct PCR amplification using primer pairs B14J06375F2/B14J05375R2 and B20J12141F2/B20J12141R2 for B14-type and B20-type, respectively. The primer pairs for B14-type and B20-type were derived from PKS51 and NRPS31, respectively. [file Image_3.tif]
